# Congenital *Candida krusei* Sepsis in an Extremely Preterm Baby: Case Report and Literature Review

**DOI:** 10.3390/antibiotics14070666

**Published:** 2025-06-30

**Authors:** Francesca Cossovel, Silvia Nider, Jenny Bua, Elena Ghirigato, Monica Piccoli, Paolo Manzoni, Laura Travan

**Affiliations:** 1 Department of Neonatology, Institute for Maternal and Child Health, IRCCS Burlo Garofolo, 34137 Trieste, Italy; silvia.nider@burlo.trieste.it (S.N.); jenny.bua@burlo.trieste.it (J.B.); laura.travan@burlo.trieste.it (L.T.); 2 Department of Medical, Surgical and Health Sciences, University of Trieste, 34127 Trieste, Italy; elena.ghirigato@burlo.trieste.it; 3 Department of Obstetrics and Gynecology, Institute for Maternal and Child Health, IRCCS Burlo Garofolo, 34137 Trieste, Italy; monica.piccoli@burlo.trieste.it; 4 Department of Pediatrics and Neonatology, University Hospital Degli Infermi, 13875 Biella, Italy; paolo.manzoni@aslbi.piemonte.it

**Keywords:** congenital candida, *Candida krusei*, preterm baby

## Abstract

A preterm neonate born at 24 + 5 weeks gestation developed congenital *Candida krusei* sepsis, diagnosed via placental culture, axillary swab, and elevated beta-glucan levels. Although initial blood cultures were negative, continuous HeRo monitoring played a crucial role in the early detection of clinical deterioration, prompting timely antifungal therapy with amphotericin B followed by micafungin. This proactive approach, combining prompt diagnosis, HeRo surveillance, and tailored treatment, ensured a favorable outcome. Our case underscores the value of HeRo monitoring as an early warning tool in managing neonatal fungal infections.

## 1. Introduction

Congenital sepsis is a life-threatening condition in neonates, caused by a variety of bacterial and fungal pathogens. Among fungal infections, *Candida* spp. is the most commonly implicated microorganism in neonatal invasive candidiasis [1] *Candida krusei* is an emerging pathogen in this context, increasingly recognized for its distinct resistance profile and the therapeutic challenges it presents [2].

One of the main difficulties in managing *C. krusei* infections is its intrinsic resistance to fluconazole and its ability to form biofilms, which further complicates treatment [3]. The pathogenesis of congenital *Candida* sepsis can involve either vertical transmission from the mother or horizontal transmission after birth. Vertical transmission occurs when *Candida* spp. ascends from the vaginal canal or crosses the placenta, leading to intrauterine infection [4]. This risk is particularly high in preterm infants, whose underdeveloped immune defenses make them highly susceptible to invasive fungal infections.

Diagnosing congenital *Candida* sepsis remains a significant challenge. Mothers may be asymptomatic or exhibit non-specific signs, and typical neonatal sepsis workups focus primarily on bacterial pathogens. Consequently, fungal infections may go unrecognized, leading to delays in appropriate antifungal treatment.

## 2. Case Presentation

A female baby was born at 24 + 5 weeks of gestation via spontaneous vaginal delivery due to unstoppable labor. Four days before the mother experienced severe abdominal pain and after MRI, the hypothesis of pyelonephritis was proposed; she was treated with antibiotic therapy (piperacillin–tazobactam, meropenem, vancomycin). Steroids as prophylaxis for respiratory distress syndrome (RDS) and magnesium sulfate were administered. Two days after delivery, the mother was eventually diagnosed with phlegmonous appendicitis.

At birth, the neonate showed good adaptation (Apgar score 8–9) and was referred to the Neonatal Intensive Care Unit on non-invasive ventilation. Approximately four hours after birth, intratracheal surfactant was administered using the IN-REC-SUR-E technique (intubation–recruitment–surfactant–extubation) due to respiratory distress with the need for FiO2 > 0.3, resulting in significant clinical improvement. Antibiotic therapy with ampicillin and tobramycin was started but discontinued after 36 h after a negative blood culture result. Fluconazole prophylaxis 3 mg/kg every 72 h was administered due to the placement of an umbilical venous catheter in an extremely low-birth-weight infant (ELBWI). On day-of-life (dol) 3, we were informed by the Microbiology Unit that *Candida krusei* was growing from the mother′s placenta culture. In the light of the extremely high-risk condition of this newborn, an axillary skin swab was performed on the baby and turned out to be positive on dol 4.

On dol 5, persistent high HeRO was detected together to a decreased reactivity. However, the neonate continued showing stable vital signs, negative reactive protein C and procalcitonin, and normal basic laboratory (WBC was 15,130/mm^3^, neutrophils 8800/mm^3^, lymphocytes 2570/mm^3^, hemoglobin 13.1 g/dL, and platelets 99,000/mm^3^ with platelet aggregates; creatinine 0.82 mg/dL, AST 57 U/L, ALT 11 U/L, GGT 85 U/L, sodium 138 mEq/L, potassium 4.3 mEq/L, total calcium 10.22 mg/dL, phosphorus 5.33 mg/dL, and total proteins 4.7 g/dL) and stable respiratory conditions under non-invasive ventilation. Given the consistent positivity for *Candida krusei* in cultures from both the mother′s placenta and the neonate′s axillary skin, systemic antifungal therapy with amphotericin B (5 mg/kg every 24 h iv) was initiated alongside empiric oxacillin and tobramycin. A sample for beta-glucan was collected, showing a markedly elevated level (1000 pg/mL; normal values 7–10), while complete blood count and inflammatory markers remained negative. Initial cultures from blood, the catheter tip, and cerebrospinal fluid were also negative.

On day-of-life (DOL) 8, considering the high beta-glucan level, a repeat blood culture was performed. This culture became positive for *Candida krusei* after only 23 h of incubation. Following this confirmation, amphotericin B was discontinued and replaced with micafungin (10 mg every 24 h iv) 48 h later, an antifungal agent known for a better penetration of both old and young biofilms [1]. This antifungal regimen was maintained for 21 days, being discontinued after obtaining two consecutively negative blood cultures together with a normalization of beta-glucan levels. The neonate maintained good general conditions throughout the course of the NICU stay and continued requiring only non-invasive respiratory support.

During antifungal therapy, the central catheter was replaced 48 h after the initiation of the treatment.

During hospitalization, end-organ fungal dissemination workup was performed weekly to rule out possible sanctuaries, and this included repeated ophthalmologic examinations and abdominal and cranial ultrasounds, all of which remained normal. At 38 weeks of corrected gestational age, a brain MRI was performed and was normal for gestational age.

The infant was weaned off respiratory support on dol 51 and achieved full feeding autonomy on dol 85, being thereafter discharged in good general condition on dol 91.

The case summary is presented in Table 1.

## 3. Discussion

Congenital candidiasis is a rare yet severe complication in neonatology, particularly affecting extremely preterm infants with underdeveloped immune defenses and disrupted skin and mucosal barriers. Among the Candida species, *Candida krusei* poses a notable clinical challenge due to its intrinsic resistance to fluconazole and its tendency to form biofilms on medical devices and epithelial surfaces [5]. Although *Candida albicans* remains the most frequently isolated species in congenital infections, an increasing proportion of neonatal fungal sepsis cases are attributed to non-albicans species, particularly in settings with widespread antifungal prophylaxis [6]. We have summarized the available literature on *Candida krusei* infections in preterm infants in Table 2 for a comprehensive reference.

This table summarizes key findings from selected case reports, case series, cohort studies, and outbreak investigations focusing on *Candida krusei* infections in preterm neonates. Both congenital (early onset) and late-onset sepsis cases are included, with details on clinical presentation, treatment, diagnostic methods, and outcomes.

The PubMed search was performed using the following string: (“*Candida krusei*” [MeSH Terms] OR “*Candida krusei*” [All Fields]) AND (“Sepsis” [MeSH Terms] OR “sepsis” [All Fields] OR “fungemia” [All Fields]) AND (“Infant, Premature” [MeSH Terms] OR “preterm infant” [All Fields] OR “premature neonate” [All Fields]) AND (“Congenital” [All Fields] OR “Vertical Transmission” [All Fields] OR “Perinatal” [All Fields]).

## 4. Epidemiology and Transmission

The vertical transmission of *Candida* can occur in utero, intrapartum, or postnatally. In the case of in utero transmission, fungal organisms may ascend from the maternal genital tract following the premature rupture of membranes or via hematogenous spread from the mother through the placenta [2,12]. *Candida krusei* colonization of the maternal genital tract is uncommon but has been reported, and it is often associated with prior antifungal exposure that selects for resistant strains [13]. While intrapartum transmission is more frequently described for *Candida albicans*, our case suggests a probable congenital (in utero) transmission route, supported by prolonged maternal antibiotic therapy, clinical presentation within the first 24 h of life, skin colonization at birth, and the absence of postnatal exposure.

According to classical diagnostic criteria for congenital infection, congenital fungal infection is supported by early neonatal evidence of infection combined with microbiological confirmation in maternal tissues. In this case, *Candida krusei* was isolated from the mother′s placenta, and the neonate showed positive fungal biomarkers and skin colonization within the first days of life, prior to blood culture positivity. These findings strongly suggest in utero or peripartum transmission. Although direct histopathological confirmation is lacking, the temporal sequence and microbiological evidence justify classifying this as a congenital infection rather than a postnatal acquisition [14,15].

The rarity of *C. krusei* as a congenital pathogen, combined with its resistance profile, elevates the importance of early diagnosis and targeted treatment. Prompt initiation of therapy is critical, as untreated or delayed fungal infections in neonates are associated with significant morbidity, including neurodevelopmental impairment, bronchopulmonary dysplasia, and mortality [16].

## 5. Clinical Presentation and Role of HeRO Monitoring

Congenital candidiasis may present as localized cutaneous disease or progress to systemic involvement, particularly in premature infants. Typical clinical signs include respiratory distress, apnea, hypotonia, poor feeding, and skin lesions such as diffuse erythematous rashes or pustules. However, these manifestations are non-specific and often indistinguishable from bacterial sepsis or other neonatal conditions [17].

In our case, the early use of HeRO (heart rate observation) monitoring provided a critical diagnostic adjunct. This system continuously evaluates heart rate variability and deceleration events to generate a predictive index of neonatal sepsis risk. Several studies have demonstrated that HeRO monitoring enables earlier detection of sepsis-related physiological changes, often before clinical deterioration becomes apparent [18]. In this instance, the elevated HeRO score preceded overt symptoms, prompting timely laboratory workup and empirical antifungal therapy. This suggests a promising role for advanced physiological monitoring in guiding early intervention, particularly when traditional clinical signs are subtle or delayed.

## 6. Diagnostic Challenges and Utility of β-D-Glucan

Confirming the diagnosis of neonatal fungal infection is fraught with difficulty. Blood cultures, though considered the gold standard, have limited sensitivity, with false negatives occurring in up to 50% of neonatal cases due to intermittent fungemia and low circulating organism load [19]. Moreover, the slow growth kinetics of fungi often delay confirmation, underscoring the need for more rapid and sensitive diagnostic tools.

In this context, (1→3)-β-D-glucan (BDG), a polysaccharide component of most fungal cell walls, has emerged as a useful biomarker. BDG assays offer a non-culture-based method to detect invasive fungal infections and have been validated in adult and pediatric critical care populations [20]. However, their application in neonates remains limited, and interpretation must be cautious due to age-specific confounders such as exposure to intravenous immunoglobulin, blood products, and mucosal colonization, all of which may cause false positive results.

In our case, the absence of a histopathological examination of the placenta and the lack of amniotic fluid cultures represent limitations. Nonetheless, the combination of clinical presentation, timing of symptom onset, and microbiological findings remains highly suggestive of congenital *Candida krusei* infection.

Despite these challenges, our patient exhibited markedly elevated BDG levels in the absence of other inflammatory markers or bacterial growth. This prompted a preemptive initiation of antifungal therapy, which was justified by positive skin cultures for *Candida krusei*. Notably, serial BDG monitoring revealed a downward trend that paralleled clinical improvement, suggesting its potential utility not only in diagnosis but also in monitoring treatment response [21]. Recent studies propose that rising or persistently elevated BDG levels in neonates should raise concern for ongoing fungal infection and prompt the re-evaluation of therapy [22].

## 7. Therapeutic Considerations and Antifungal Strategy

The management of congenital *C. krusei* infection necessitates a prompt initiation of systemic antifungal therapy with agents active against this species. Amphotericin B deoxycholate has long been the cornerstone of neonatal antifungal therapy due to its broad-spectrum activity and fungicidal effects. However, its nephrotoxicity, electrolyte imbalances, and infusion-related adverse events pose significant risks, particularly in preterm neonates with fragile renal function and fluid balance [23].

In our case, treatment was initiated with amphotericin B, but due to its more effective penetration into both old and young biofilms, a transition to micafungin was undertaken. Echinocandins, including micafungin and caspofungin, inhibit β-(1,3)-D-glucan synthase, impairing fungal cell wall synthesis. They have demonstrated excellent efficacy against *C. krusei*, and unlike amphotericin B, possess activity against biofilm-embedded fungal cells, which is particularly relevant in the context of central lines and endovascular infection [24]. While echinocandin use in neonates has historically been limited due to sparse pharmacokinetic data, recent studies support their safety and efficacy when appropriately dosed. High-dose micafungin regimens have been evaluated in neonatal trials, showing favorable therapeutic outcomes without significant hepatotoxicity or hematologic toxicity [2]. Notably, the use of micafungin in neonates remains off-label in many countries, including those following EMA and FDA guidance due to limited formal approval; however, clinical guidelines such as those from the Infectious Diseases Society of America (IDSA) and the European Society for Paediatric Infectious Diseases (ESPID) endorse its use in this population under specific conditions [25]. Our patient responded well to micafungin, with a resolution of signs of sepsis and normalization of BDG levels.

## 8. Multidisciplinary Management and Outcome

The successful management of neonatal fungal infections, especially those caused by rare and resistant organisms, requires close collaboration among neonatologists, infectious disease specialists, microbiologists, and pharmacists. In this case, the decision-making process was facilitated by prompt laboratory communication, real-time monitoring, and a proactive approach to diagnostic and therapeutic escalation. Multidisciplinary discussions guided antifungal selection, the monitoring of renal and hepatic function, and decisions about treatment duration and follow-up.

Despite the traditionally poor prognosis associated with *C. krusei* and other non-albicans species in preterm infants, our patient had a favorable outcome. This likely reflects a combination of factors, including extremely early detection, appropriate therapeutic adjustments, and consistent follow-up. A growing body of literature supports the notion that early, aggressive intervention in fungal infections—particularly in high-risk neonates—can significantly improve outcomes [26].

## 9. Implications for Practice and Future Directions

This case contributes to a limited but growing body of evidence on congenital *C. krusei* infections. It reinforces the need for vigilance in recognizing early signs of sepsis in neonates, particularly when standard bacterial cultures are negative. BDG testing, although not yet standard in all neonatal intensive care units, represents a promising adjunct for early detection and therapy guidance.

There is also a clear need for additional research on antifungal pharmacodynamics and pharmacokinetics in neonates, particularly for newer agents such as echinocandins and azoles like voriconazole or isavuconazole. Expanding clinical experience with echinocandins, supported by prospective multicenter trials, could help standardize dosing protocols and safety monitoring in this vulnerable population [27].

Finally, this case underscores the importance of maternal screening, especially in high-risk pregnancies with a prolonged rupture of membranes or prior antifungal exposure. While routine screening for fungal colonization is not currently recommended, targeted screening in selected scenarios may warrant reconsideration, especially in settings where resistant Candida species are prevalent.

## 10. Conclusions

To our knowledge, this is the first reported case of congenital, vertically transmitted *Candida krusei* infection in an extremely preterm neonate, successfully managed with early antifungal therapy guided by advanced monitoring and biomarker evaluation. It highlights the diagnostic challenges of neonatal fungal sepsis, the therapeutic potential of echinocandins in this setting, and the vital role of multidisciplinary collaboration. Our experience supports a further incorporation of non-culture diagnostics like BDG and an expanded use of real-time physiological monitoring to improve early recognition and outcomes in neonatal fungal infections.

## Figures and Tables

**Table 1:** Timeline of clinical events, laboratory findings, diagnostic procedures, HeRO monitoring, and therapeutic interventions throughout the hospital stay of the preterm neonate with congenital *Candida krusei* infection. The table highlights key milestones from birth to discharge, including early microbiological detection, antifungal therapy adjustments based on culture results, and progressive clinical improvement with the normalization of laboratory markers and successful respiratory weaning.

Day of Life (DOL)	Clinical Events	Laboratory Findings/Diagnostics	Therapeutic Interventions	HeRO Score
Birth (DOL 0)	Female preterm neonate born at 24 + 5 weeks, Apgar 8–9	—	Non-invasive ventilation; intratracheal surfactant (IN-REC-SUR-E technique) due to respiratory distress (FiO_2_ > 0.3)	Baseline
DOL 0–1	Stable respiratory status	—	Antibiotics started: ampicillin + tobramycin; fluconazole prophylaxis started due to UVC placement	Stable
DOL 3	Microbiology report: *Candida krusei* from mother′s placenta	—	—	—
DOL 4	Neonate axillary swab positive for *Candida krusei*	—	—	—
DOL 5	Persistent high HeRO score; decreased reactivity	WBC 15,130/mm^3^; neutrophils 8800/mm^3^; lymphocytes 2570/mm^3^; Hb 13.1 g/dL; platelets 99,000/mm^3^ (with aggregates); creatinine 0.82 mg/dL; AST 57 U/L; ALT 11 U/L; GGT 85 U/L; Na 138 mEq/L; K 4.3 mEq/L; Ca 10.22 mg/dL; P 5.33 mg/dL; total proteins 4.7 g/dL; PCR negative; PCT negative; beta-glucan 1000 pg/mL	Amphotericin B started; empiric oxacillin and tobramycin continued	Elevated
DOL 6–7	Stable vital signs and respiratory condition	Blood cultures negative	Continued antifungal and antibiotic therapy	Decreasing
DOL 8	Repeat blood culture positive for *Candida krusei* (23 h)	Elevated beta-glucan level persists	Amphotericin B discontinued 48 h after culture; started micafungin 10 mg/kg/day IV	—
DOL 9–29	Clinical improvement; monitoring for fungal dissemination	Serial blood cultures negative; beta-glucan decreasing	Continued micafungin for 21 days	Normalizing
~DOL 11	Central catheter replaced	—	Catheter replacement	—
Weekly (DOL 8–91)	Ophthalmologic exams, abdominal and cranial ultrasounds normal	—	Continued supportive care	—
DOL 51	Weaning off respiratory support	—	Respiratory support discontinued	—
DOL 85	Full feeding autonomy achieved	—	—	—
DOL 91	Discharged in good general condition	—	—	—
38 weeks corrected GA	Brain MRI normal for gestational age	—	—	—

**Table 2:** Summary of published studies on congenital and late-onset neonatal sepsis caused by *Candida krusei* in preterm infants [7,8,9,10,11].

Study	Year	Study Design	Population	Candida Species	Onset Type	Key Findings	Treatment	Symptoms at Onset	Diagnostic Method	Outcome
Wanjari et al.	2008	Case Report	28-week preterm infant	*Candida krusei*	Congenital (early onset, day 2)	Blood culture positive on day 2; resistant to fluconazole; sensitive to amphotericin B	Amphotericin B	Fever	Blood culture	Successful treatment
Amaral-Lopes et al.	2012	Case Series	3 neonates (27–28 weeks GA)	*Candida krusei*, *C. albicans*	Congenital (early GI symptoms)	Early GI symptoms; early treatment initiation	Fluconazole	Abdominal distension, bilious regurgitation	Blood culture	Favorable outcomes in two; late fungal infection in one
Tiraboshi et al.	2010	Case Report	27-week preterm infant	*Candida krusei*, *C. albicans*	Likely congenital (chorioamnionitis)	Blood cultures positive; chorioamnionitis as trigger	Not specified	Not specified	Blood culture	Favorable outcome
Gauteng, South Africa	2014	Outbreak Study	589 infants	*Candida krusei*	Late onset (nosocomial)	Total of 48 cases of candidemia; associated with NEC, LBW, TPN, blood transfusions	Not specified	Not specified	Blood culture	Ongoing outbreaks despite interventions
India	2019	Cohort Study	551 VLBW and ELBW infants	*C. albicans*, *C. tropicalis*, *C. parapsilosis*, *C. krusei*, *C. glabrata*	Late onset (hospital acquired)	Total of 64 invasive Candida sepsis cases; 6 *C. krusei*	Not specified	Not specified	Blood, urine, CSF cultures	High mortality (55%); no fluconazole prophylaxis

## Data Availability

We confirm that all relevant data supporting the findings are included in the article, and no further datasets are available.
